# The Woman's Hospital Association

**Published:** 1857-10

**Authors:** 


*The Woman's Hospital Association.*—We have before us several pamphlets giving an account of the workings of this admirable charity, and of the action of the Legislature of New York, making it a state institution. A brief sketch of its history may prove interesting to our readers.

Dr. J. Marion Sims, of Alabama, has become most favorably known to the medical profession by his great improvements in the operation for vaginal fistulas. This distressing and disgusting disease which embitters the whole life of the unfortunate female who suffers from it, has hitherto been an *opprobrium medicorum.* The most eminent surgeons of France, Germany, and this country have wholly and entirely failed to accomplish any satisfactory cure by operation. Dr. Sims, however took the matter in hand, and after repeated failures and numerous attempts, at last succeeded in effecting a complete restoration of the parts which had suffered from this frightful malady. Ill health compelled him to leave his southern home, and he selected New York as a residence. There he interested some wealthy and philanthropic ladies who formed themselves into an association for the establishment of a hospital exclusively appropriated to the treatment of female diseases. The Woman's Hospital is the result of their well directed efforts to relieve the sufferings of their sex.

By the annual report, we learn that in 1856, 98 patients were treated at this institution, with the most gratifying success. Hitherto, the association has kept up the Hospital by private contributions, but now, by Act of Legislature, passed last April, it has become a state institution. To what extent it is to derive aid from the state, remains to be seen; it is to be hoped, however, that a spirit of liberality will actuate the authorities in dealing with the institution.

We call the attention of our readers to this excellent institution and its skillful attending surgeon, in the hope that the information we have thus given, may be of service in directing some suffering woman to a source whence she may obtain relief, elsewhere denied her.

